# LentiGlobin Administration to Sickle Cell Disease Patients: Effect on Serum Markers and Vaso-Occlusive Crisis

**DOI:** 10.7759/cureus.51881

**Published:** 2024-01-08

**Authors:** Harendra Kumar, Vagisha Sharma, Sohmika S Wadhwa, Diksha Mahadeva Gowda, Sharanya Kaushik, Andrew M Joseph, Monica Karas, Jonathan Quinonez, Anthony Furiato

**Affiliations:** 1 Medicine and Surgery, Dow University of Health Sciences, Karachi, PAK; 2 Medicine, Vardhman Mahavir Medical College and Safdarjung Hospital, New Delhi, IND; 3 Medicine, Government Medical College and Hospital, Nagpur, IND; 4 Psychiatry, Kempegowda Institute of Medical Sciences, Bengaluru, IND; 5 Internal Medicine, Bangalore Medical College, Bengaluru, IND; 6 Osteopathic Medicine, Nova Southeastern University Dr. Kiran C. Patel College of Osteopathic Medicine, Davie, USA; 7 Osteopathic Medicine/Neurology, Larkin Community Hospital Palm Springs Campus, Hialeah, USA; 8 Addiction Medicine, Brandon Regional Hospital, Brandon, USA; 9 Emergency Medicine, Brandon Regional Hospital, Brandon, USA

**Keywords:** blood disorders, gene therapy, vaso-occlusive crisis, sickle cell disease, lentiglobin

## Abstract

LentiGlobin, an innovative gene therapy, introduces a modified beta-globin gene that yields an anti-sickling hemoglobin variant. It boosts total hemoglobin levels, mitigates hemolysis, curtails inflammation, and addresses iron overload by reducing transfusion requirements. These changes, in turn, provide insights into disease mechanisms and treatment outcomes. Alterations in serum markers, such as hemoglobin levels and inflammatory biomarkers, can illuminate the therapeutic effectiveness of LentiGlobin and its impact on mitigating complications such as vaso-occlusive crises. Therefore, the purpose of this narrative review is to discuss the effects of LentiGlobin administration on diverse serum biomarkers and its correlation with vaso-occlusive crises in individuals with sickle cell disease (SCD).

## Introduction and background

Vaso-occlusive events (VOE) that painfully recur are a hallmark of sickle cell disease (SCD). The available supportive therapies include pain medications and blood transfusions. The standard of care also includes hydroxyurea, which has been shown to reduce sickle cell crises and increase survival, although it is frequently underused [1]. Hydroxyurea can control sickle cell disease but not cure it [2,3]. A potentially curative therapeutic approach is HLA-matched sibling allogeneic hematopoietic stem cell transplantation [4,5]. However, this approach is limited because only around 18% of patients have donors that match their HLA, which increases the likelihood of graft-versus-host disease, graft rejection, and the risk of transplant-related death. HLA-matched sibling allogeneic hematopoietic stem cell transplantation is also primarily recommended for younger patients [6]. 

LentiGlobin, an innovative gene therapy, is another approach recently introduced as a possible management of vaso-occlusive crises. LentiGlobin introduces a modified beta-globin gene that yields an anti-sickling hemoglobin variant [4]. It boosts total hemoglobin levels, mitigates hemolysis, curtails inflammation, and addresses iron overload by reducing transfusion requirements [4]. These changes, in turn, provide insights into disease mechanisms and treatment outcomes [4]. Alterations in serum markers, such as hemoglobin levels and inflammatory biomarkers, can illuminate the therapeutic effectiveness of LentiGlobin and its impact on mitigating complications such as vaso-occlusive crises [4].

A patient with transfusion-dependent thalassemia was recently reported to show the first evidence of conversion to transfusion independence using a lentiviral vector. This patient produced a marked and anti-sickling T87Q-globin gene variation endogenously. BB305 is a lentiviral vector that encodes a modified globin gene that produces the antisickling hemoglobin, HbAT87Q, and is used in gene therapy for sickle cell disease (bb1111; lovotibeglogene autotemcel) [4].

Several other gene therapy trials are ongoing for transfusion-dependent thalassemia and sickle cell disease. In another trial using the GLOBE lentiviral vector, encouraging results were recently reported in children with transfusion-dependent thalassemia [7]. The ability of lentiviral vectors to transduce and gene-correct nondividing cells gives them a significant advantage over retroviral vectors. Since most human hematopoietic stem cells are non-dividing, lentiviruses allow for greater effectiveness in gene transfer and expression [7-9]. A phase 1 clinical trial in Paris was started on a LentiGlobin vector harboring a human globin gene with a single amino acid modification at position 87 of the amino acid of the globin protein sequence [8]. It has been demonstrated that Hb87 behaves like fetal hemoglobin and preferentially obstructs sickle cell development [9]. After a single administration of LentiGlobin, most red blood cells continued to produce HbAT87Q, which reduced hemolysis and completely resolved severe vaso-occlusive episodes [2].

The quantity of LentiGlobin infused was directly related to other transplantation outcomes [10], such as growth factor demand, the length of stay in the hospital, and neutrophil and platelet recovery [11,12]. Therefore, the purpose of this narrative review is to discuss the effects of LentiGlobin administration on diverse serum biomarkers and its correlation with vaso-occlusive crises in individuals with SCD.

## Review

Pathogenesis of vaso-occlusive crisis

Sickle cell disease is a syndrome of diseases with profound multiorgan effects. It is an autosomal recessive inherited blood disorder caused by a point mutation at the sixth codon of the beta-globulin gene. The mutation causes a single amino acid replacement of a negatively charged glutamine by a neutral valine, which reduces or destroys normal beta-globulin production. When present in a deoxygenated state, the mutant sickle beta-globulin subunits (HbS) of the hemoglobin tetramers can polymerize and cause the erythrocytes to undergo a rapid but reversible shape change. They become sickle- or crescent-shaped, which makes the erythrocytes rigid [13]. These sickled erythrocytes, on re-oxygenation, restore their normal shape. The repeated process of sickling and re-sickling makes the erythrocyte membrane less flexible, resulting in intravascular hemolysis or extravascular removal by the reticuloendothelial system [14].

The manifestations of sickle cell disease can be acute or chronic. The acute complications are severe anemia, infections, and vaso-occlusive crises. The chronic complications are osteoporosis/avascular necrosis, neurological deficits/seizures, pulmonary hypertension, cardiomyopathy, impaired renal function, retinal detachment, chronic leg ulcers, and growth retardation in children [15].

A vaso-occlusive crisis is a common and significant complication of sickle cell disease. The triggers can be infection, stress, or exposure to the cold, resulting in the erythrocytes sickling. The mechanism of the vaso-occlusive crisis involves the polymerization of deoxygenated hemoglobin and sickled erythrocytes. These cells become rigid and do not flow easily through the microcirculation. Subsequently, the sickled erythrocytes have increased adhesion to vascular endothelial cells and leucocytes, activating the hemostatic mechanism. The adhesion process contributes to microvascular occlusion, impaired blood flow, and tissue hypoxia, resulting in pain and organ damage [16]. Ischemia-reperfusion injury and inflammation create a vicious cycle, triggering the production of reactive oxygen species (ROS) that cause oxidative stress, release pro-inflammatory cytokines, and further exacerbate endothelial dysfunction [17].

LentiGlobin's mechanism of action and rationale

The definitive therapies for SCD include regular blood exchange transfusions and long-term use of disease-modifying drugs (hydroxyurea), which reduce the symptoms but do not eliminate or halt the disease's progression. Gene therapy by autologous transplantation is a promising curative alternative, carrying lower transplant-related risks (immunosuppression and GVHD) for SCD patients [17].

LentiGlobin is a new gene therapy for certain blood disorders, especially SCD and beta-thalassemia. This gene therapy involves the extraction of hematopoietic stem cells from the patient's bone marrow or peripheral blood. The extracted autologous cells are transduced with the BB305 lentiviral vector (modified virus) carrying the functional beta-globulin gene, which produces antisickling hemoglobin. After the modification, the modified stem cells are infused back into the patient's body. These cells eventually differentiate into different blood cells, including erythrocytes containing the corrected beta globulin. The new cells produce healthy hemoglobin A (HbA) instead of abnormal HbS [16]. As a result, the risk of red cell distortion and complications is significantly reduced.

Serum markers and their significance in sickle cell disease 

There is a wide range of serum markers that will be abnormal in the setting of SCD and other pathological conditions [5]. These markers are presented along with a brief description in Tables 1-4. The primary diagnostic tools for managing SCD are hemoglobin and fetal hemoglobin levels, along with reticulocyte and white blood cell counts that can be identified in serum (serum levels are listed in Tables 1-4).

**Table 1:** Hemolysis-related markers used to assess and monitor hemolysis in individuals with sickle cell disease. LDH: lactate dehydrogenase [5,13,14].

Marker	Expected serum level	Description
Hemoglobin	Decrease	The most useful biomarker in SCD. A result of increased red blood cell destruction.
Bilirubin	Elevated	Due to the breakdown of heme molecules from hemoglobin
LDH	Elevated	LDH is released when red blood cells rupture
Reticulocyte count	Elevated	An increase in the production of new red blood cells by the bone marrow is often in response to the higher turnover resulting from hemolysis
Haptoglobin	Decreased	Haptoglobin binds to hemoglobin released from ruptured red blood cells, preventing its accumulation in the bloodstream
Hemopexin levels	Decreased	Another protein that binds to free heme, the component of hemoglobin, decreased hemopexin levels can be associated with increased hemolysis
Free hemoglobin	Presence in bloodstream	Due to ruptured red blood cells, can contribute to oxidative stress and inflammation
Indirect bilirubin	Elevated	Results from the breakdown of hemoglobin and increased hemolysis
Urinary hemoglobin	Elevated	Hemoglobin released from lysed red blood cells can be filtered by the kidneys and excreted in the urine

**Table 2:** Inflammatory markers that assist with monitoring the state of inflammation and endothelial dysfunction and guide treatment strategies. CRP: C-reactive protein; IL-6: interleukin-6; IL-1β: interleukin-1-beta [5,13,14].

Marker	Expected serum level	Description
C-reactive protein (CRP)	Elevated	General marker of inflammation
Interleukins (IL-6, IL-1β)	Elevated	Pro-inflammatory cytokines
Tumor necrosis factor-a	Elevated	Pro-inflammatory cytokines
Erythrocyte sedimentation rate	Elevated	Non-specific marker of inflammation
Leukocyte count	Elevated	Indicates inflammation

**Table 3:** Endothelial activation markers used collectively to provide insights into the state of inflammation and endothelial dysfunction in individuals with sickle cell disease. sICAM-1: soluble intercellular adhesion molecule-1; sVCAM-1: soluble vascular cell adhesion molecule-1; vWF: von Willebrand factor; ET-1: endothelial-1; TM: thrombomodulin [5,13,14].

Marker	Expected serum level	Description
sICAM-1	Elevated	ICAM-1 is involved in the adhesion of white blood cells to the endothelium
sVCAM-1	Elevated	Endothelial activation and potential inflammation
E-selectin and P-selectin	Elevated	Cell adhesion molecules are expressed on activated endothelial cells. Indicate endothelial activation and potential for cell adhesion
vWF	Elevated	Suggest endothelial dysfunction and are associated with an increased risk of vaso-occlusive events
ET-1	Elevated	ET-1 is a peptide that causes vasoconstriction and endothelial dysfunction
TM	Elevated	Present on the surface of endothelial cells and helps regulate coagulation

**Table 4:** Endogenous vasodilator markers that are disrupted in SCD leading to impaired endothelial function and increased vasoconstriction. NO: nitric oxide; PGI2: prostacyclin-I2; 5-HT: serotonin [5,13,14].

Marker	Expected serum level	Description
Nitric oxide (NO)	Decreased	A potent vasodilator produced by endothelial cells
Prostacyclin (PGI2)	Decreased	Promotes vasodilation and inhibits platelet aggregation
Serotonin (5-HT)	Elevated	A vasoactive molecule that can have both vasoconstrictive and vasodilatory effects. In SCD, serotonin is released during vaso-occlusive events and contributes to vasoconstriction.

LentiGlobin's effects on vaso-occlusive crisis

The erythrocytes derived from the gene therapy-modified stem cells produce functional hemoglobin, which has a higher affinity for oxygen than HbS and does not cause the characteristic sickling seen in sickle cell disease. These changes prevent the abnormal shape of red blood cells that can lead to blockages in blood vessels and subsequent vasocclusive crises. However, the precise mechanism for the LentiGlobin-mitigated reduction in vaso-occlusive crises is complex and may involve multiple factors that can influence the disease process.

Gene therapy has allowed patients to discontinue or significantly reduce blood transfusions and remain clinically well [17]. An interim analysis showed that one-time treatment with LentiGlobin resulted in sustained benefits for sickle cell disease and resolution of severe vaso-occlusive complications [16].

Changes in specific markers, such as hemoglobin levels, erythropoietin, and reticulocyte counts, can indicate successful gene therapy and improvements in hemoglobin production. After LentiGlobin infusion, there was an increased production of HbA, a lower total hemoglobin level, and reduced hemolytic markers. LentiGlobin administration may trigger an immune response due to the viral vectors used in gene delivery. Therefore, monitoring markers of inflammation, such as C-reactive protein (CRP) and interleukins, aids in assessing the degree of immune reaction and potential adverse effects associated with the therapy. Abnormal levels of liver enzymes, bilirubin, and other biochemical markers may indicate potential hepatotoxicity or other systemic reactions that must be addressed promptly. Monitoring changes in markers such as ferritin level is crucial in evaluating iron overload, a common concern in patients with hemoglobinopathies [17-19].

Clinical trials and findings

Several clinical trials and studies have been conducted to evaluate the safety and efficacy of LentiGlobin gene therapy for SCD patients. The most prominent and instructive trials are given below.

HGB-205

A phase 1/2 study of the LentiGlobin BB305 drug product in patients with transfusion-dependent β-thalassemia and severe SCD [18]. This study was the first to demonstrate the proof of concept for LentiGlobin gene therapy in SCD, showing sustained production of anti-sickling hemoglobin (HbA T87Q) and reduction of hemolysis and VOEs in three treated patients within the age group 5-35 years old [19-21].

HGB-206

A phase 1/2 study of the LentiGlobin BB305 drug product in patients with severe SCD [22]. This study enrolled 40 patients in three cohorts (A, B, and C) with different drug product manufacturing processes and myeloablative conditioning regimens. One cohort (group C) showed that LentiGlobin gene therapy resulted in near-pancellular expression of HbA T87Q, reduced sickling and hemolysis, increased total hemoglobin levels, and resolved serious VOEs and chronic pain [23,24].

HGB-210

A Phase 3 study of the LentiGlobin BB305 drug product in patients with severe SCD [6]. This study is currently recruiting patients and aims to enroll 45 participants. The primary outcome is the proportion of patients who achieve a reduction of at least 60% in the annualized rate of VOEs two years after LentiGlobin infusion compared to the two years before treatment [6].

LentiGlobin gene therapy for SCD has been shown to affect various serum markers that reflect the pathophysiology of the disease. Some of the main findings are given below.

Hemoglobin levels

LentiGlobin gene therapy increased the total hemoglobin levels in SCD patients by producing HbA T87Q, which reduced the proportion of HbS and improved oxygen delivery. In the HGB-206 study, one cohort (group C) had a median increase of 5.2 g/dL in HbA T87Q levels and a median decrease of 4.0 g/dL in HbS levels at their last visit [24].

Hemolysis Markers

LentiGlobin gene therapy reduced hemolysis by decreasing the sickling propensity and increasing RBC survival. In the HGB-206 study, one cohort (group C) had a median reduction (76%) in LDH, a marker of hemolysis, and a median increase in haptoglobin (94%), a protein that binds to free hemoglobin, at their last visit [4,24].

Inflammation Markers

LentiGlobin gene therapy may also reduce inflammation, a chronic state of immune activation, and tissue damage in SCD by decreasing the exposure of RBC membrane antigens and reducing the adhesion of RBCs to endothelial cells. In the HGB-206 study, one cohort (group C) had a median reduction of 32% in C-reactive protein (CRP), a marker of inflammation, at their last visit [24].

The reduction or elimination of VOEs, episodes of acute pain caused by the obstruction of blood flow due to sickled RBCs, is one of the most important outcomes of LentiGlobin gene therapy for SCD. Age groups involved in the trials vary based on study design and eligibility criteria. The HGB-205 study enrolled individuals aged 5-35, with a median age of 16 for β-thalassemia and 13 for SCD [1]. Meanwhile, the HGB-206 study included those aged 12-50, with a median age of 25 for group C [7]. Currently recruiting, the HGB-210 study targets patients aged 2-50 [3]. These ranges aim to encompass pediatric and adult severe SCD cases suitable for potential gene therapy benefits. Triggers for sickling, such as low oxygen, dehydration, stress, or high altitude, cause abnormal hemoglobin S (HbS) to form rigid sickle-shaped cells, blocking vessels and leading to pain and tissue damage [11]. Psychological stress can activate the sympathetic nervous system, constricting vessels [11]. The exact mechanisms vary, possibly involving genetics, inflammation, or endothelial dysfunction. Identification and avoidance of triggers are crucial, along with prompt medical attention if sickness occurs. The adverse effects of LentiGlobin gene therapy are mainly tied to cell collection, conditioning, and infusion [6]. Common effects, like reduced blood cell levels or mucositis, are usually manageable. However, serious effects such as infection or graft-versus-host disease may arise, requiring vigilant monitoring during long-term follow-ups, as observed in the HGB-206 study, where no hematologic cancer cases were noted over 37.6 months [7]. VOEs are associated with significant morbidity, mortality, and healthcare utilization in SCD patients. In preliminary trials, LentiGlobin gene therapy reduces VOEs by increasing the production of HbA T87Q, which prevents or reverses sickle cell disease and improves blood rheology. In the HGB-206 study, one cohort (group C) had a median annualized rate of VOEs requiring hospitalization or emergency department visits of 3.2 per year before and 0 after treatment [24]. Moreover, none of the group C patients experienced acute chest syndrome (ACS), a life-threatening complication of VOEs that involves lung injury and respiratory failure, after LentiGlobin infusion [24]. Additionally, LentiGlobin gene therapy improved patient-reported pain intensity, as measured by the Patient Reported Outcomes Measurement Information System (PROMIS)-57 questionnaire. Group C patients had a median reduction of 3 points in their pain intensity score at their last visit compared to baseline [23,24].

Potential mechanisms for changes in serum markers

LentiGlobin, a groundbreaking gene therapy, holds promise for revolutionizing the management of beta-thalassemia and sickle cell disease by introducing a modified beta-globin gene (β A-T87Q) into hematopoietic stem cells. The resultant hemoglobin variant (HbA T87Q) possesses unique anti-sickling properties, compensating for the malfunctioning or absent beta-globin chains inherent to these diseases [1]. The intricate interplay of this therapy with serum markers brings to light several pivotal mechanisms that underlie its effects.

LentiGlobin administration is anticipated to increase total hemoglobin levels, thereby mitigating anemia. The production of HbA T87Q fosters the formation of functional tetramers alongside alpha-globin chains, culminating in enhanced oxygen delivery to tissues [21,22]. A second mechanism involves the reduction of hemolysis and the subsequent release of free hemoglobin and heme into circulation. Achieved by curbing the propensity for sickling and extending the lifespan of red blood cells (RBCs), this mechanism is proposed to decrease markers such as lactate dehydrogenase (LDH) and bilirubin [21,22].

Furthermore, LentiGlobin has the potential to alleviate inflammation and oxidative stress. By diminishing the exposure of RBC membrane antigens and reducing the adhesion of RBCs to endothelial cells, the therapy could curtail immune activation and avert tissue damage [3]. Additionally, LentiGlobin may curtail iron overload and the concomitant risk of organ damage. This gene therapy addresses pivotal sources of iron accumulation in these disorders via its role in reducing transfusion requirements and intestinal iron absorption.

The implications of these serum marker changes extend beyond the molecular realm, offering valuable insights into disease pathogenesis and treatment outcomes. An elevation in total hemoglobin levels and HbA T87Q concentrations may serve as indicators of engraftment degree and gene-modified HSC transduction efficiency. This observation also signals rectifying the globin chain imbalance, a hallmark of these conditions [2]. Conversely, reducing hemolysis markers such as LDH and bilirubin suggests improvements in RBC morphology and function, coinciding with a diminished frequency of VOEs and hemolytic crises [22-24].

The gene therapy's multifaceted effects extend to haptoglobin and inflammation markers. Increased haptoglobin levels symbolize the restoration of haptoglobin-hemoglobin complexes, crucial in mitigating heme-mediated oxidative damage and enhancing nitric oxide scavenging, a vasodilatory function. Concurrently, a decrease in inflammation markers, notably C-reactive protein (CRP) and interleukin-6 (IL-6), implies the attenuation of chronic inflammation, potentially reducing endothelial dysfunction, thrombotic events, and organ fibrosis [4,25,26].

The potential for LentiGlobin to combat iron overload is of equal significance, as reflected by the decrease in serum ferritin levels. Reducing iron burden offers a protective shield against the development of cardiomyopathy, liver cirrhosis, endocrinopathies, and susceptibility to infections. The multifaceted serum marker changes brought about by LentiGlobin administration illuminate its complex mechanisms of action in beta-thalassemia and SCD (Table 5). These changes signify therapeutic efficacy and provide a nuanced understanding of disease pathogenesis, thus offering a transformative perspective on treatment outcomes.

**Table 5:** Potential mechanisms of serum marker changes following LentiGlobin administration in beta-thalassemia and sickle cell disease. HbA: hemoglobin A; RBC: red blood cell; LDH: lactate dehydrogenase; VOE: vaso-occlusive events; CRP: C-reactive protein; IL-6: interleukin-6 [22,23].

Mechanism	Serum marker changes	Implications
Increased total hemoglobin and HbA T87Q levels	Increase in hemoglobin levels	Assessment of gene therapy efficacy
	Rise in HbA T87Q levels	Correction of globin chain imbalance
Reduced hemolysis and RBC survival	Decrease in LDH levels	Improved RBC morphology and function
	Lowered bilirubin levels	Reduced VOEs
Attenuated inflammation and oxidative stress	Decrease in CRP levels	Improved endothelial function
	Reduction in IL-6 levels	Mitigated thrombosis and organ damage
Lower iron overload and organ damage risk	Decrease in serum ferritin levels	Minimized iron-induced organ pathologies
	Decreased transfusion requirements	Lessened risk of cardiomyopathy
Restored haptoglobin-hemoglobin complexes	Increase in haptoglobin levels	Protection against heme-mediated damage - improved vasodilation through nitric oxide scavenging

Implications for clinical practice and future research

LentiGlobin is a promising gene therapy that has yielded substantial improvement in clinical outcomes and the quality of life in patients with severe SCD. However, some considerations exist for incorporating LentiGlobin into the current SCD management, including the availability and accessibility of lentigine, a complex and costly procedure requiring specialized facilities. It may not be widely available or accessible to all patients who need it, especially in low-resource settings where SCD is prevalent [23].

Therefore, strategies to increase the availability and accessibility of LentiGlobin, such as developing simplified and standardized protocols, optimizing the manufacturing and delivery processes, reducing costs and barriers, and expanding global collaborations and partnerships, are needed to ensure that more patients can benefit from this innovative therapy [24]. Another consideration is the eligibility and selection of patients for LentiGlobin, which is based on several criteria, such as the severity of SCD, the availability of a suitable donor, the presence of comorbidities or complications, and the patient's preference and consent. LentiGlobin is not a one-size-fits-all solution for all SCD patients, and some patients may not be eligible or willing to undergo this procedure. Therefore, carefully evaluating and counseling patients for LentiGlobin and providing alternative or complementary therapies, such as hydroxyurea, transfusions, or pain management, are essential to optimizing individualized care and shared decision-making for SCD patients. A third consideration is the safety and efficacy of LentiGlobin, which are still under investigation in ongoing clinical trials and studies.

LentiGlobin involves gene modification and transplantation techniques, which carry potential risks and uncertainties, such as insertional mutagenesis, graft failure or rejection, infection, or an immune reaction [4,24-26]. Therefore, rigorous monitoring and evaluation of LentiGlobin safety and efficacy outcomes, as well as reporting and managing any adverse events or complications, are crucial to ensuring the quality and reliability of this therapy.

## Conclusions

In summary, LentiGlobin shows promise as a gene therapy for sickle cell disease, offering potential improvements in clinical outcomes and quality of life by generating anti-sickling hemoglobin. However, challenges such as complexity, cost, and accessibility, as well as associated risks like insertional mutagenesis and conditioning regimen side effects, underscore the need for further research to enhance the safety and efficacy of LentiGlobin gene therapy for widespread application in SCD patients.
